# Supplementary material for: Mapping immunodominant sites on the MERS-CoV spike glycoprotein targeted by infection-elicited antibodies in humans
Source: Cell Rep. Author manuscript; Available in PMC 2025 Aug 11. (PMC12338757; doi:10.1016/j.celrep.2024.114530)
Supplement: SuppFigures [file NIHMS2094784-supplement-SuppFigures.pdf]

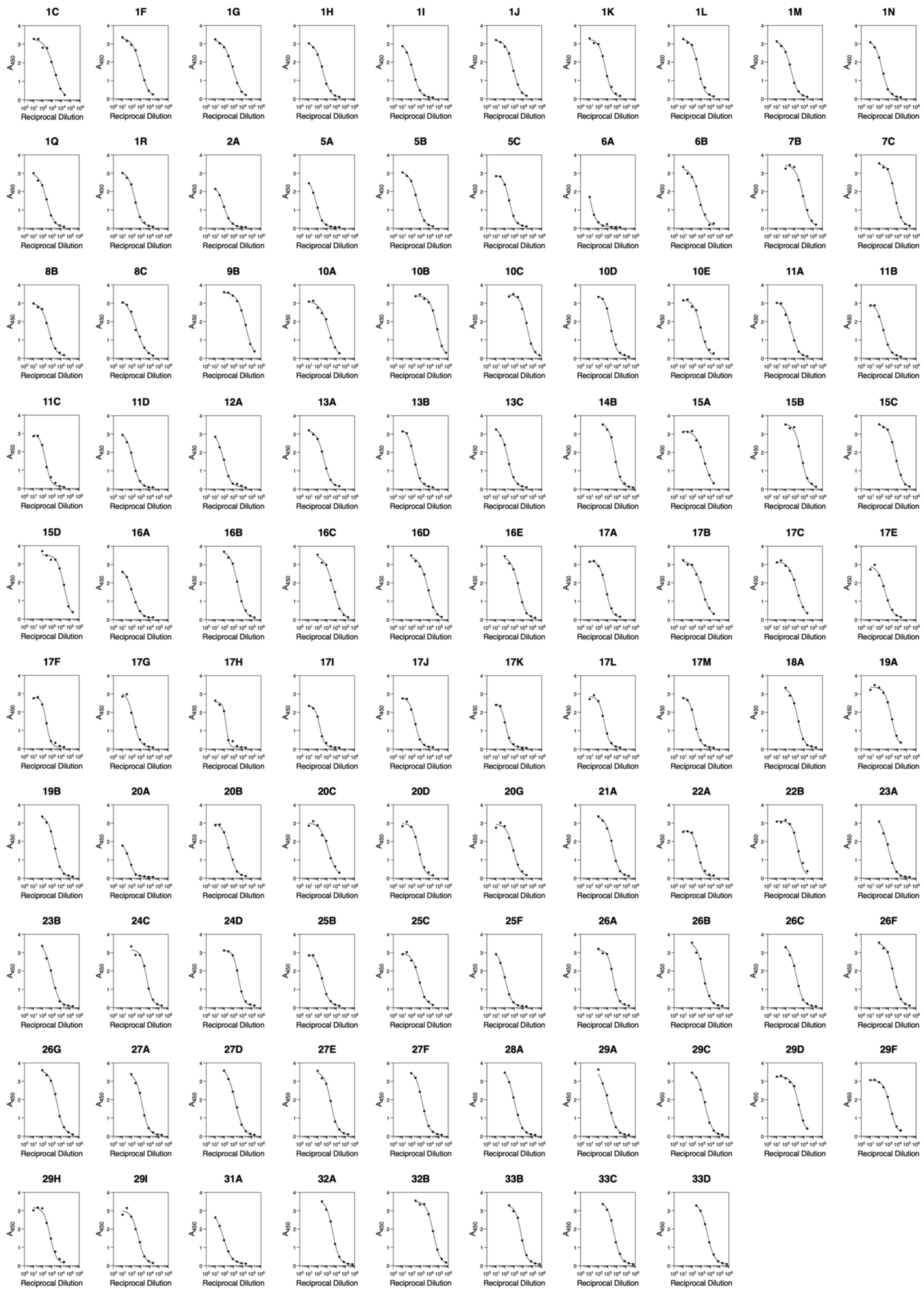
1C
1F
1G
1H
1I
1J
1K
1L
1M
1N
1Q
1R
2A
5A
5B
5C
6A
6B
7B
7C
8B
8C
9B
10A
10B
10C
10D
10E
11A
11B
11C
11D
12A
13A
13B
13C
14B
15A
15B
15C
15D
16A
16B
16C
16D
16E
17A
17B
17C
17E
17F
17G
17H
17I
17J
17K
17L
17M
18A
19A
19B
20A
20B
20C
20D
20G
21A
22A
22B
23A
23B
24C
24D
25B
25C
25F
26A
26B
26C
26F
26G
27A
27D
27E
27F
28A
29A
29C
29D
29F
29H
29I
31A
32A
32B
33B
33C
33D
A_{450}
Reciprocal Dilution

Figure S1. Evaluation of MERS-CoV S-directed plasma IgG binding titers, related to Figure 1. Dose-response curves of plasma IgG binding to prefusion-stabilized MERS-CoV EMC/2012 2P S for each of the 98 samples analyzed in this study by ELISA. Data are presented for one representative biological replicate. At least two biological replicates each using a unique batch of MERS-CoV S were conducted for each sample.

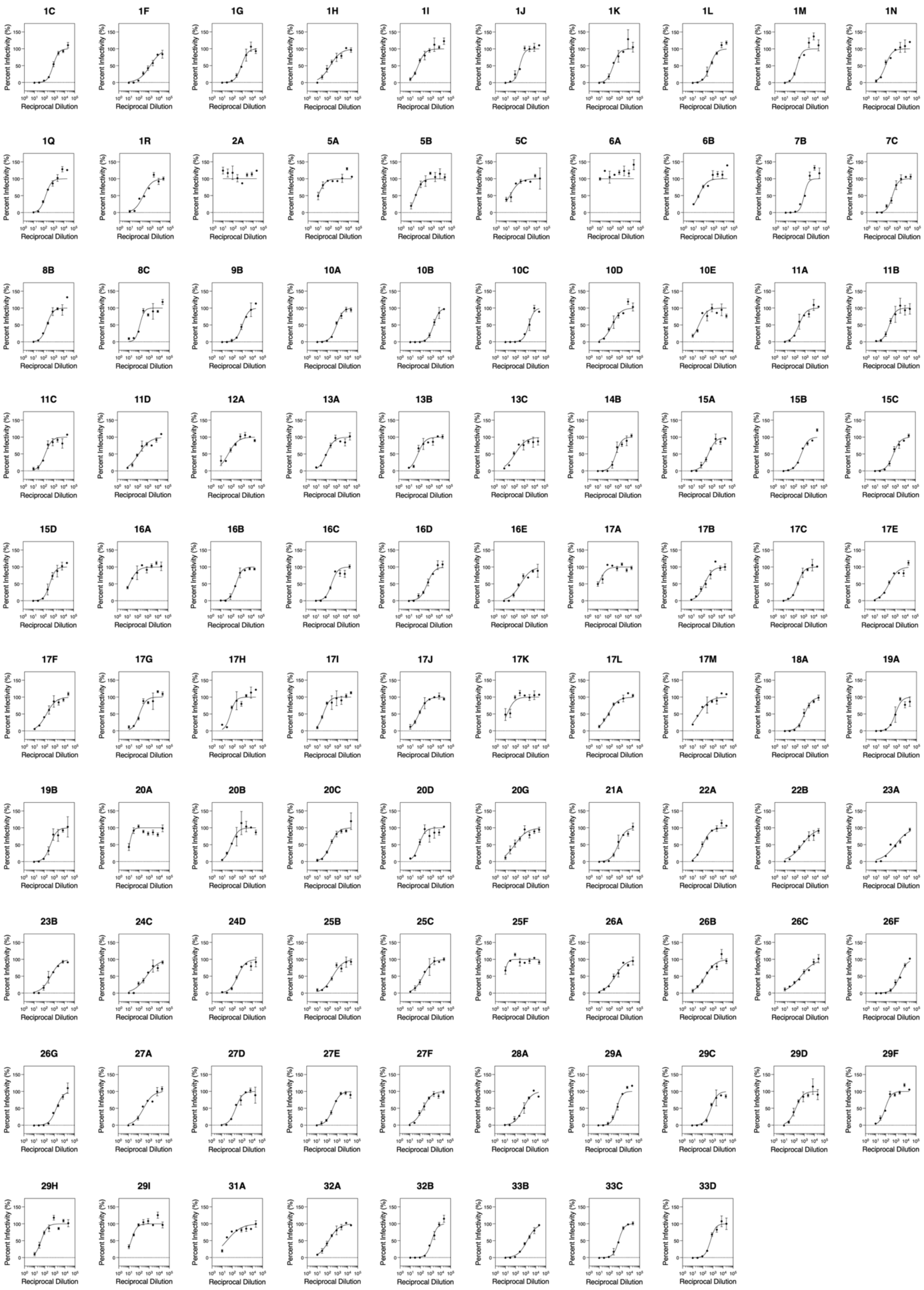
1C
1F
1G
1H
1I
1J
1K
1L
1M
1N
1Q
1R
2A
5A
5B
5C
6A
6B
7B
7C
8B
8C
9B
10A
10B
10C
10D
10E
11A
11B
11C
11D
12A
13A
13B
13C
14B
15A
15B
15C
15D
16A
16B
16C
16D
16E
17A
17B
17C
17E
17F
17G
17H
17I
17J
17K
17L
17M
18A
19A
19B
20A
20B
20C
20D
20G
21A
22A
22B
23A
23B
24C
24D
25B
25C
25F
26A
26B
26C
26F
26G
27A
27D
27E
27F
28A
29A
29C
29D
29F
29H
29I
31A
32A
32B
33B
33C
33D
Percent Infectivity (%)
Reciprocal Dilution

Figure S2. Evaluation of plasma neutralization potency against MERS-CoV EMC/2012, related to Figure 1. Dose-response curves of plasma neutralization of VSV pseudotyped with MERS-CoV EMC/2012 S for the 98 samples analyzed in this study. Data are presented as the mean ± standard error of two technical replicates from one representative biological replicate. At least two biological replicates with two technical replicates were completed for each sample using two distinct batches of pseudovirus.

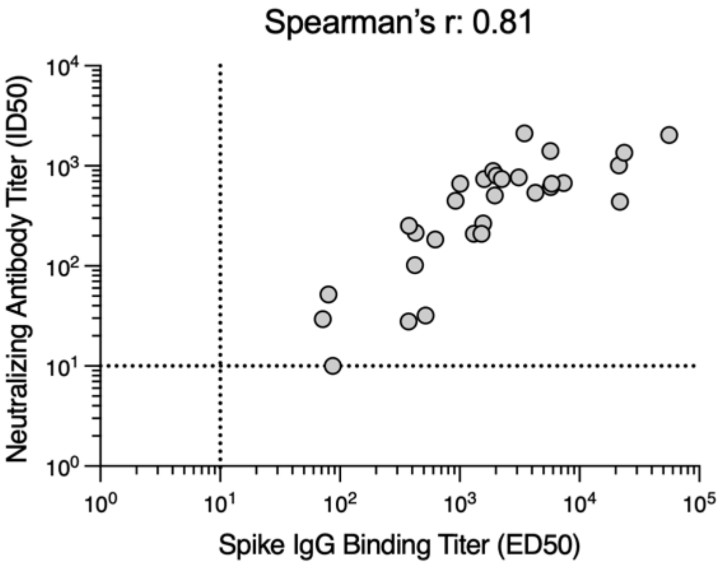

Figure S3. Correlation analysis of S-directed IgG binding titers and neutralizing antibody titers, related to Figure 1. The sample with highest S IgG binding titer per individual was included in the analysis. The limits of detection (ED_{50} or ID_{50}: ≤ 10) are indicated with dashed lines.

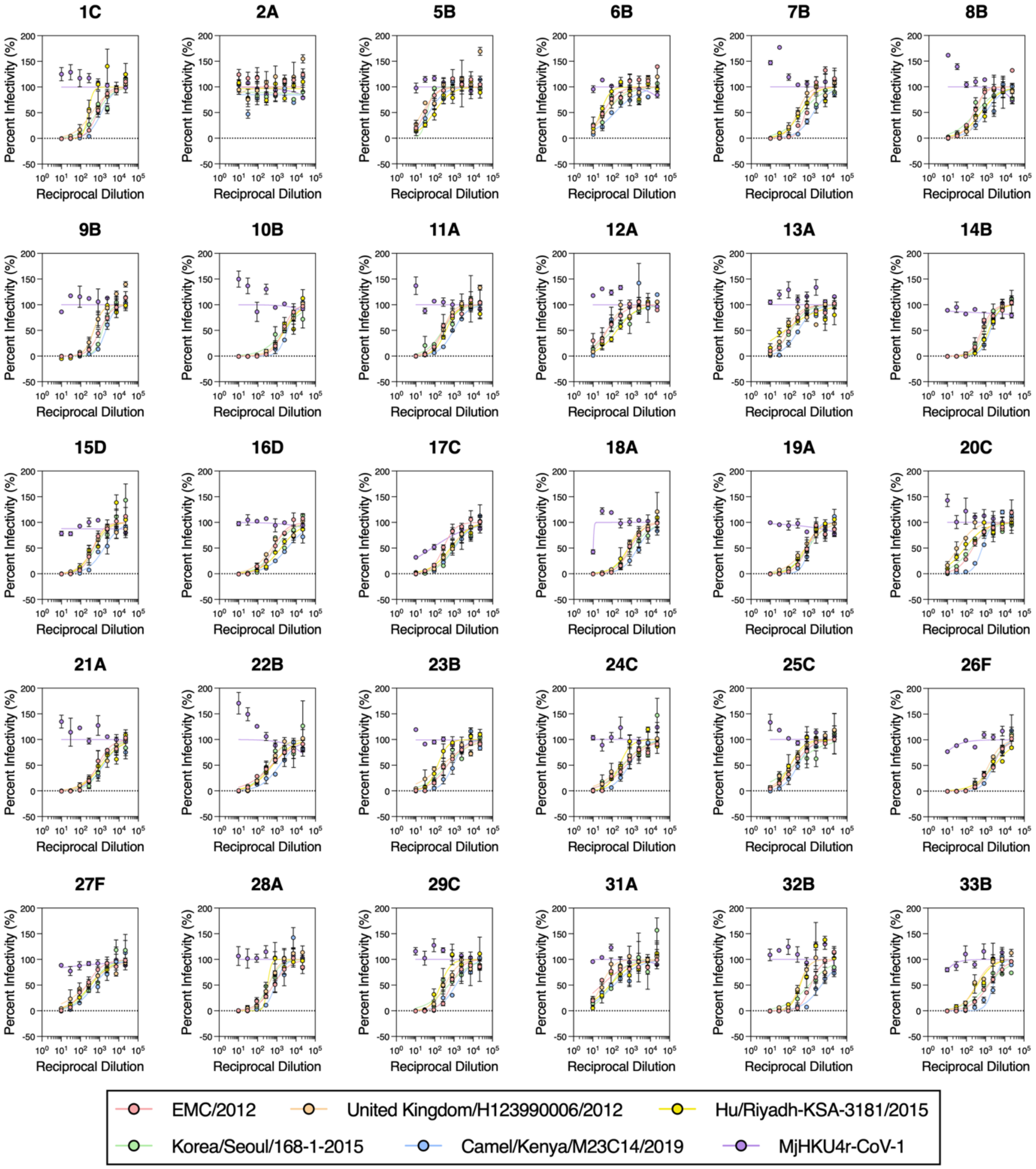

Figure S4. Evaluation of plasma neutralization potency against MERS-CoV variants and MjHKU4r-CoV-1, related to Figure 1. Dose-response curves for each of the 30 plasma samples included in the analysis using VSV pseudotyped with the indicated MERS-CoV variant or MjHKU4-CoV-1 S protein. Data are presented as mean ± standard error from one representative biological replicate. At least two biological replicates, each with two technical replicates, were conducted for each sample and each variant tested using unique batches of pseudovirus.

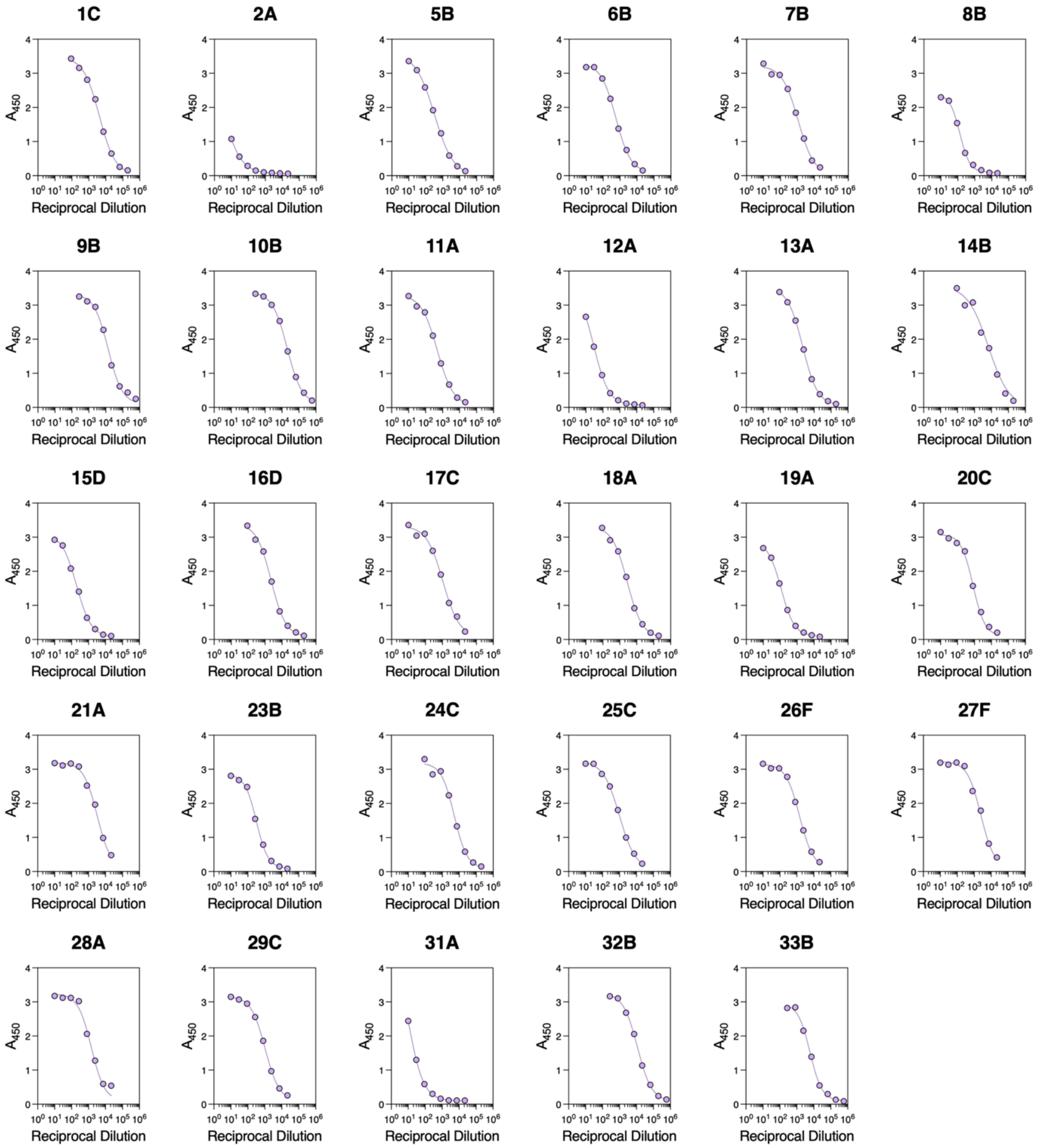

Figure S5. Evaluation of MERS-CoV S_1-directed plasma IgG binding titers, related to Figure 2. Dose-response curves of plasma IgG binding to MERS-CoV S_1 analyzed by ELISA. The sample with the highest S IgG binding titer per individual was included in the analysis. Individual 22 was excluded due to sample volume limitations. Data obtained from one representative biological replicate are presented. Two biological replicates using distinct batches of S_1 protein were completed for each sample.

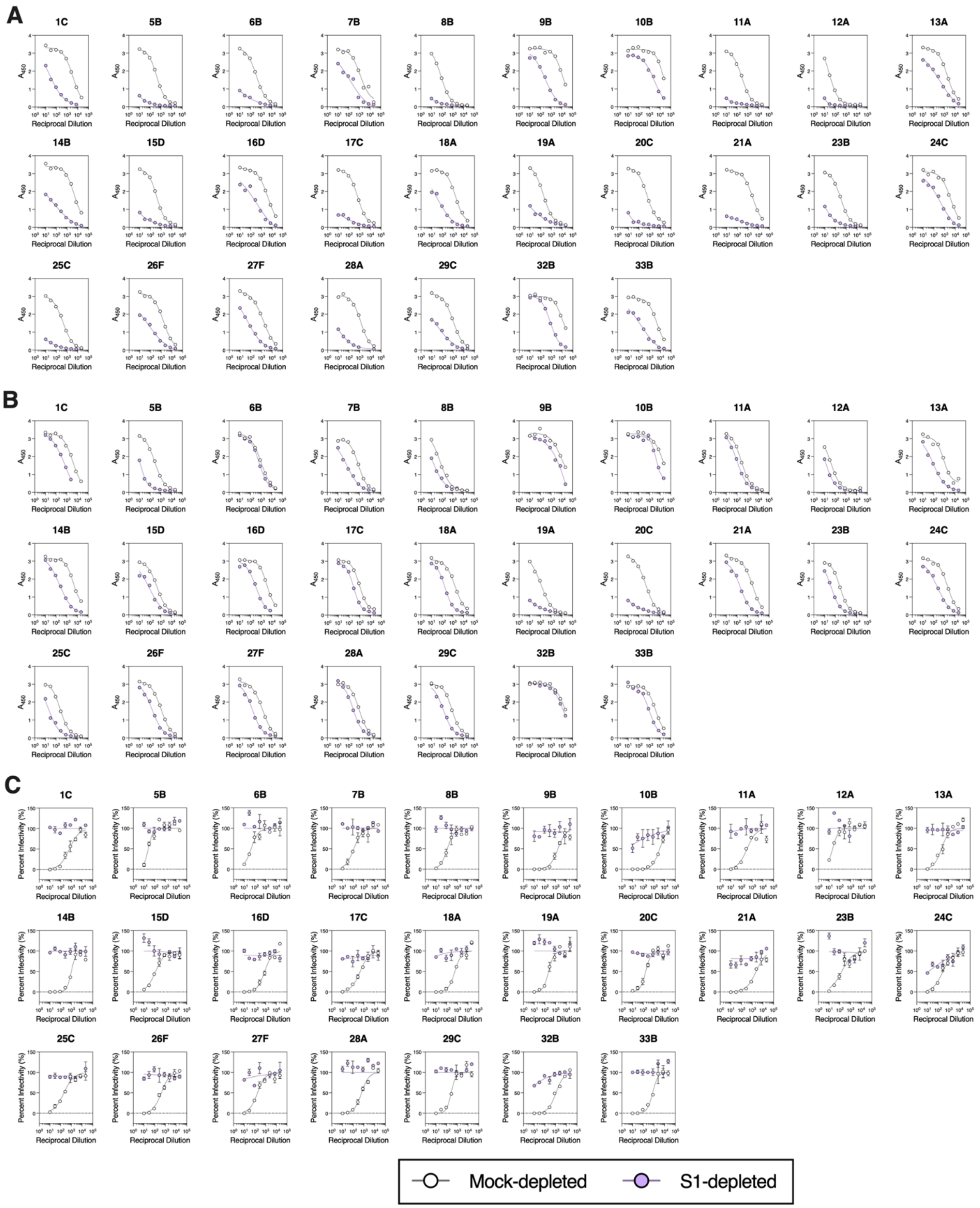

Figure S6. Evaluation of plasma IgG binding and neutralizing activity following depletion of MERS-CoV S_1-directed antibodies, related to Figure 2. A-B) Dose-response curves of S_1-(A) and S-directed (B) IgG binding for each of the 27 samples included in the analysis upon mock- or S_1-depletion analyzed by ELISA using MERS-CoV EMC/2012 S_1 or prefusion-stabilized 2P S. **C)** Dose-response curves of plasma

neutralization of VSV pseudotyped with MERS-CoV EMC/2012 S for the mock- and S_1-depleted plasma samples. Neutralization data are presented as mean ± standard error for the two technical replicates conducted. Data presented are from one representative biological replicate. Two independent biological replicates were performed using distinct batches of S_1 protein for the antibody depletions as well as unique batches of S_1 and S glycoproteins and pseudovirus for the ELISAs and neutralization assays, respectively.

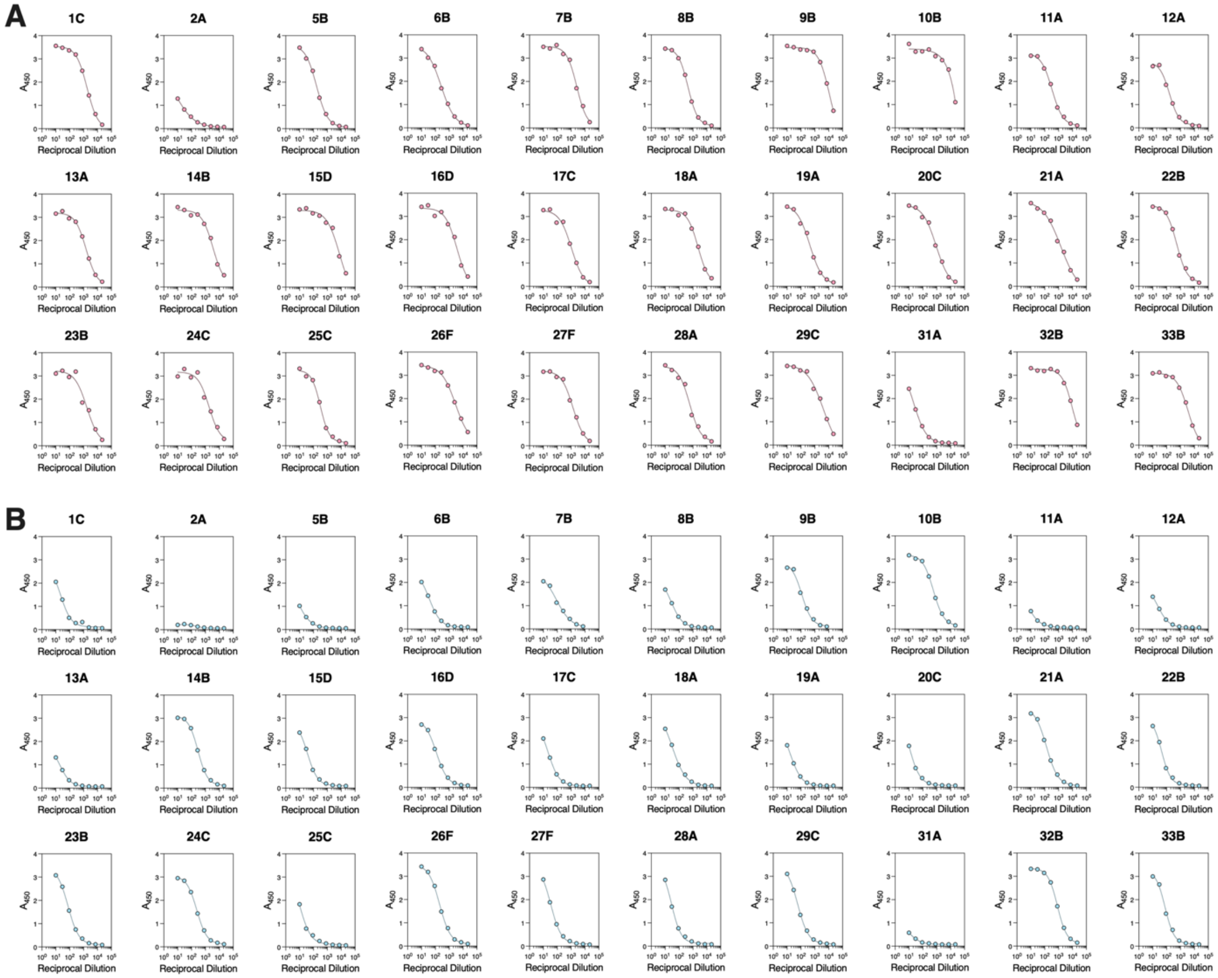

Figure S7. RBD and NTD IgG binding curves, related to Figure 3. A-B) Dose-response curves of RBD-(A) and NTD-directed (B) IgG binding for each of the 30 samples included in the analysis analyzed by ELISA using recombinantly expressed MERS-CoV EMC/2012 RBD or NTD. Data are presented from one representative biological replicate. At least two biological replicates were conducted using distinct batches of RBD or NTD.

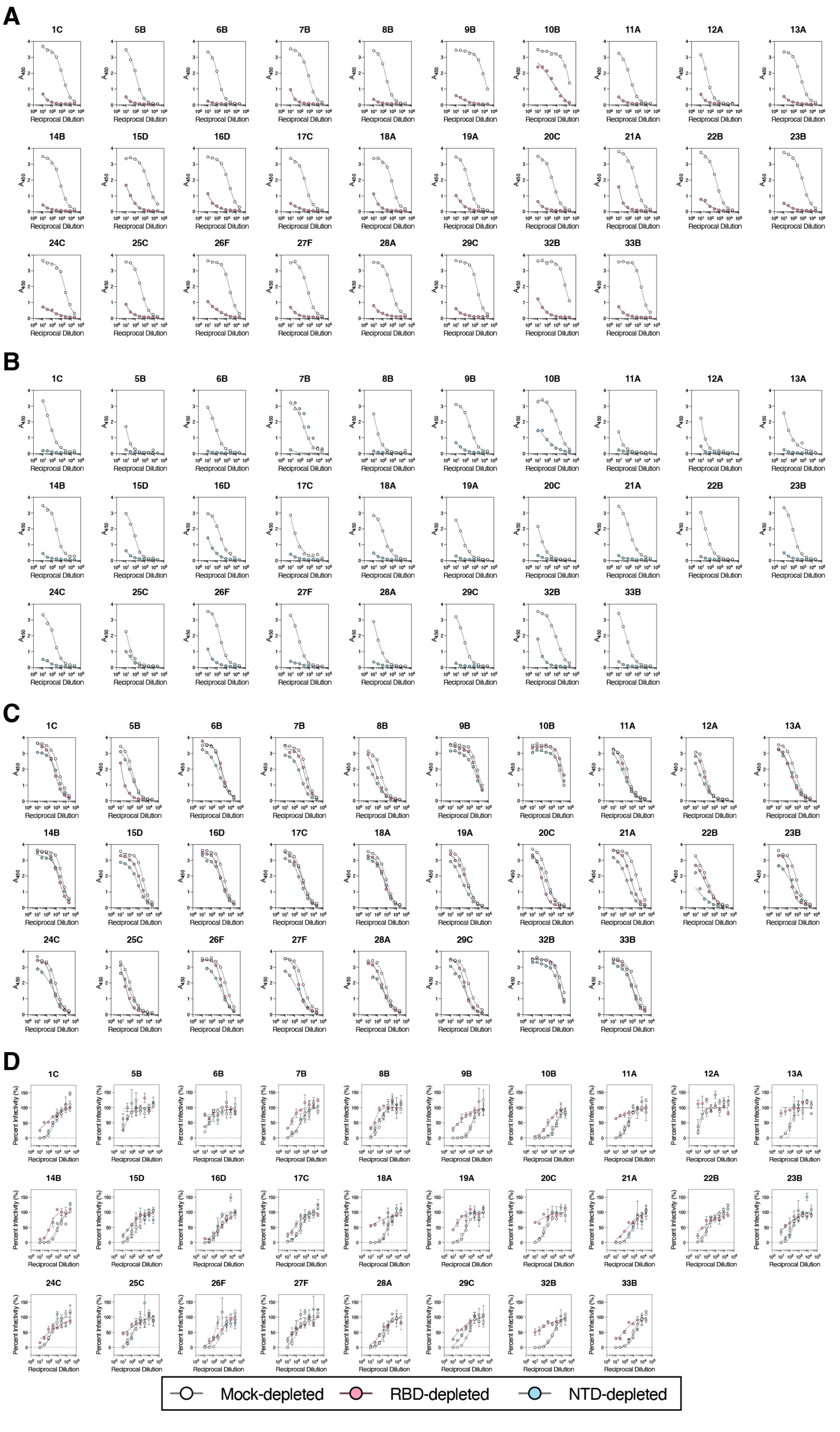
A
B
C
D
1C 5B 6B 7B 8B 9B 10B 11A 12A 13A
14B 15D 16D 17C 18A 19A 20C 21A 22B 23B
24C 25C 26F 27F 28A 29C 32B 33B
A450
Percent Infectivity (%)
Reciprocal Dilution
Mock-depleted
RBD-depleted
NTD-depleted

Figure S8. IgG binding and neutralization curves following depletion with MERS-CoV RBD and NTD, related to Figure 3. A) RBD IgG binding dose-response curves for each of the 28 samples included in the ELISA analysis after mock-depletion and depletion of RBD-directed antibodies. **B)** NTD IgG binding dose-response curves for each of the 28 samples included in the ELISA analysis after mock-depletion and depletion of NTD-directed antibodies. **C)** S IgG binding dose-response curves for each of the 28 samples included in the ELISA analysis after mock-depletion, depletion of RBD-directed or of NTD-directed antibodies. **D)** Dose-response curves for plasma neutralization of MERS-CoV S VSV after mock-depletion, depletion of RBD-directed or of NTD-directed antibodies. Neutralization data are presented as mean ± standard error for the two technical replicates conducted. Data presented are from one representative biological replicate. Two independent biological replicates were performed using distinct batches of RBD and NTD proteins for the antibody depletions as well as unique batches of RBD, NTD, and spike proteins and pseudovirus for the ELISAs and neutralization assays, respectively.

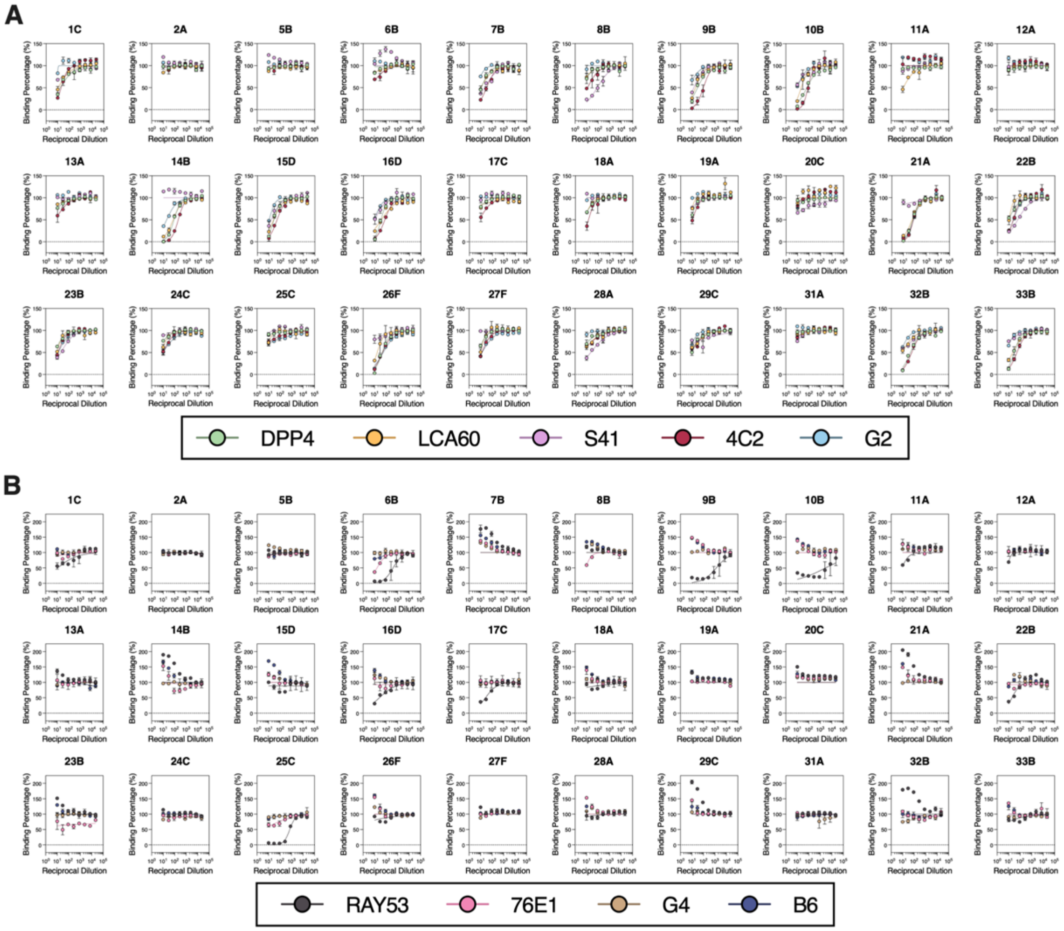

Figure S9. Competition ELISA curves, related to Figure 4. A-B) Competition ELISA curves for the 30 plasma samples analyzed using the indicated S_1-directed (A) and S_2-directed (B) monoclonal antibodies and prefusion-stabilized MERS-CoV EMC/2012 2P S. Data are presented as mean ± standard error for two technical replicates from one representative biological replicate. Two independent biological replicates using unique batches of biotinylated spike protein as well as monoclonal antibodies were performed for each sample.
